# Admixture and Genetic Diversity Distribution Patterns of Non-Recombining Lineages of Native American Ancestry in Colombian Populations

**DOI:** 10.1371/journal.pone.0120155

**Published:** 2015-03-16

**Authors:** Catarina Xavier, Juan José Builes, Verónica Gomes, Jose Miguel Ospino, Juliana Aquino, Walther Parson, António Amorim, Leonor Gusmão, Ana Goios

**Affiliations:** 1 Instituto de Investigação e Inovação em Saúde, Universidade do Porto, Porto, Portugal; 2 Institute of Molecular Pathology and Immunology of the University of Porto (IPATIMUP), Porto, Portugal; 3 Institute of Legal Medicine, Innsbruck Medical University, Innsbruck, Austria; 4 Instituto de Biología, Universidad de Antioquia, Medellín, Colombia; 5 Laboratorio Genes Ltda, Medellín, Colombia; 6 DNA Diagnostic Laboratory (LDD), State University of Rio de Janeiro (UERJ), Rio de Janeiro, Brazil; 7 Eberly College of Science, Penn State University, University Park, PA, United States of America; 8 Faculdade de Ciências da Universidade do Porto, Porto, Portugal; Universitat Pompeu Fabra, SPAIN

## Abstract

Genetic diversity of present American populations results from very complex demographic events involving different types and degrees of admixture. Through the analysis of lineage markers such as mtDNA and Y chromosome it is possible to recover the original Native American haplotypes, which remained identical since the admixture events due to the absence of recombination. However, the decrease in the effective population sizes and the consequent genetic drift effects suffered by these populations during the European colonization resulted in the loss or under-representation of a substantial fraction of the Native American lineages. In this study, we aim to clarify how the diversity and distribution of uniparental lineages vary with the different demographic characteristics (size, degree of isolation) and the different levels of admixture of extant Native groups in Colombia. We present new data resulting from the analyses of mtDNA whole control region, Y chromosome SNP haplogroups and STR haplotypes, and autosomal ancestry informative insertion-deletion polymorphisms in Colombian individuals from different ethnic and linguistic groups. The results demonstrate that populations presenting a high proportion of non-Native American ancestry have preserved nevertheless a substantial diversity of Native American lineages, for both mtDNA and Y chromosome. We suggest that, by maintaining the effective population sizes high, admixture allowed for a decrease in the effects of genetic drift due to Native population size reduction and thus resulting in an effective preservation of the Native American non-recombining lineages.

## Introduction

**Fig 1:**
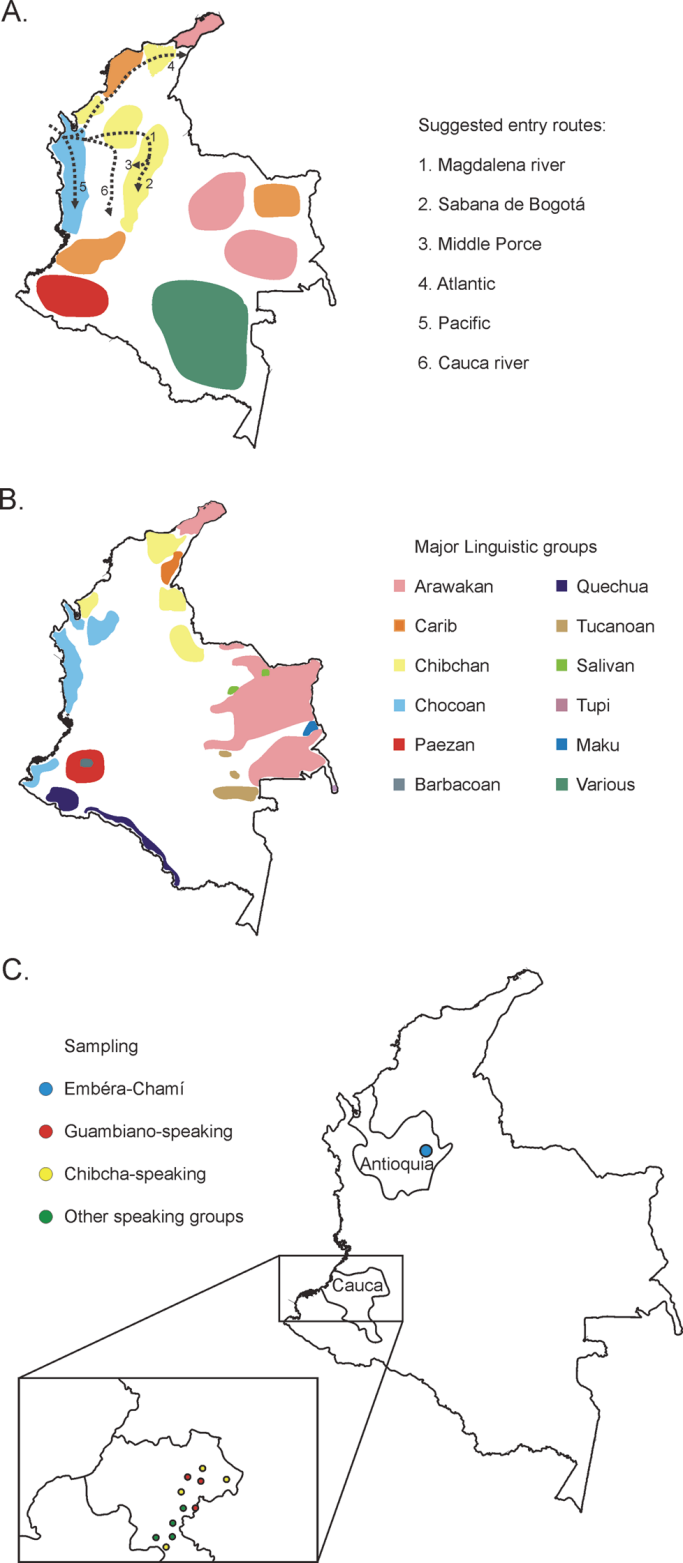
Map of Colombia showing the geographic distribution of the major linguistic groups at the time of the Spanish colonization (a) and in the present day (b), the major entrance routes in Colombia during the peopling of South America (dashed arrows in a) and the location of the population groups analysed in this study (c). Data used to produce this figure are included in references [5–9].

Colombia was the major entrance point into South America during the peopling of the continent by the Paleoindians [1]. From here, two major migratory routes took place resulting in the colonization of South America: (a) along the Pacific coastline and the Andean regions and (b) towards the Amazonian plains (Fig. 1) [2–4].

The demography of Native ethnic groups in Colombia endured several changes since pre-colonial times, through the Spanish domain and into present day. Several linguistic groups co-exist in Colombia since before the Spanish colonization, some of the most relevant being: the Chibchan, Carib and Arawakan, in the Atlantic coast, Chocoan in the Pacific coast and Paezan, Barbacoan and Quechua in the Southern Andean region (Fig. 1) [5–7]. The colonization by the Spaniards reshaped Native slavery in 1503, and later introduced the African slave trade, leading to severe alterations in the demography of Native groups [5–7], so that the genetic background of Colombia results from a mixture of Native American, African and European contributions. Furthermore, in the initial stages of the colonization, only a minority of European (around 10%) passengers and African slaves to America were women [10–12], leading to a differential male/female admixture ratio visible in several Colombian regions[12,13]. Nowadays Colombia presents 87 recognized ethnic groups and even though Castilian is the official language in Colombia, there are still 64 indigenous languages spoken throughout the country [7]. The Native groups inhabit mainly rural zones and small villages of the country or indigenous reserves; however there is a small minority that lives in the cities normally due to the lack of lands in the reserves or to difficulties in re-adapting to the social/cultural indigenous lifestyle [7].

Colombia has been the target of several forensic and anthropological genetic studies [2–4,12–30]. The genetic composition of Colombian populations has shown to be consistent with that from other South American populations with clear admixed profiles [12]. The proportions of Native American (NAM), African (AFR) and European (EUR) lineages vary throughout the country [10,13,25,27,31], and several small, isolated populations maintain a non-admixed NAM or AFR composition [28,32]. Different sizes and degrees of isolation of the settlements could have led to differential drift effects after the admixture events and to fluctuations of the frequencies of parental lineages in the admixed populations.

In the present work, we have focused on understanding how the different characteristics (source populations, size and degree of isolation, assessed by the differential input of maternal and paternal non-NAM lineages) of the remnant Native groups in Colombia have shaped the diversity and distribution of their uniparentally transmitted lineages, and whether the autosomal ancestry informative insertion-deletion (AIM-InDel) markers confirm these patterns.

## Materials and Methods

### Ethics Statement

All samples involved in this study are long-lasting anonymized DNA extracts previously obtained from healthy individuals, from paternity cases, under informed consent for research purposes. The current study was approved by the institutional review board of IPATIMUP, and conducted in accordance with the ethical principles of the 2000 Helsinki Declaration of the World Medical Association (http://www.uma.net/e/policy/b3.htm).

### Sampling

Sampling was carried out in two distinct Colombian regions (Fig. 1). In the Northern Colombian province of Antioquia, we sampled 38 unrelated Emberá-Chamí individuals from a single settlement—La Po, Segovia (07°04′47″N 74°42′06″W)—whose language belongs to the Emberá group of the Chocoan linguistic family that is considered close to the Chibcha linguistic group [8,33,34]. This is a small, isolated native reserve from which nearly all possible unrelated individuals were sampled. In Cauca (South Colombia), we sampled 58 unrelated individuals from different municipalities (S6 Table) and different ethnic and linguistic groups, collected during paternity casework in the Genes Laboratory. The major linguistic groups here represented are: (a) Guambiano (n = 33), classified as belonging to the Barbacoan family with an undefined affiliation [35,36] and composed by individuals from Guambiano and Coconuco ethnic groups and (b) Chibcha, composed by individuals belonging to the Nasa ethnic group (n = 14), whose common dialect, Nasa Yuwe, is becoming less and less used by younger generations. This dialect is classified as a Paezan language and inserted in the Chibchan-Paezan macro group [5,8,33,35]. The linguistic groups of the remaining samples from Cauca are Quechua (n = 6), Emberá (n = 1) or unidentified (n = 4).

#### MtDNA amplification and sequencing

The analysis of the mtDNA control region was performed by PCR/sequencing reactions. The entire control region was amplified using the primers L15997 (5’-CACCATTAGCACCCAAAGCT-3’) and H639 (5’-GGGTGATGTGAGCCCGTCTA-3’) [37,38]. The PCR conditions were as follow: 15 minutes at 95°C for initial denaturation, followed by 35 cycles of 30 seconds at 94°C, 90 seconds at 58°C and 90 seconds at 72°C, followed by a final extension for 10 minutes at 72°C. Amplified mtDNA was purified with ExoSAP-IT (GE Healthcare) under the following conditions: 15 minutes at the temperature of 37°C followed by 15 minutes at 80°C. Sequencing reaction was performed using the Big Dye Terminator v3.1 cycle Sequencing Kit (Applied Biosystems) and the primers L15997 and L16555 (5’-CCC ACA CGT TCC CCT TAA AT-3’) for forward sequencing and H016 (5’-CCC GTG AGT GGT TAA TAG GGT-3’) and H639 for reverse sequencing [37,38]. Sequencing reaction conditions were: initial denaturation of 2 minutes at 96°C, followed by 35 cycles of 15 seconds at 96°C and 2 minutes at 60°C, and a final extension for 10 minutes at 60°C. A final purification with Sephadex (Illustra Sephadex DNA Grade; GE Healthcare) was performed before the samples were run in the automatic sequencer ABI 3130XL (Genetic Analyzer 3000, Applied Biosystems).

#### Y chromosome genotyping

Samples for Y chromosome typing include the subset of male individuals from the total group that was typed for mtDNA. From all male samples, those that belong to Native American haplogroup Q were included in a previous study by Roewer et al. [39]. The remaining samples, which had the ancestral state on Y-SNPs present in Multiplex Q, were typed for additional SNPs through a hierarchical approach using multiplex PCR and single-base extension by SNaPshot (the full set of 35 Y-SNPs investigated in the total sample is indicated in S1 Fig.). First, the Multiplex 1 was typed in all non-haplogroup Q samples as described by Brion et al. [40] but excluding M22 marker. Samples showing the derived alleles at SRY10831.1 and M213 and the ancestral allele at M9, for the Y-SNPs included in Multiplex 1, were further typed using the Multiplex 2 [40]. Those samples showing the derived allele at just the SRY10831.1 locus in Multiplex 1 were further typed using the Multiplex E as previously described [41]. All the multiplexes were analysed as described in Gomes et al. [41]. All samples were also typed for 17 Y-STRs using the AmpFlSTR Yfiler kit following the manufacturers’ recommendations (Applied Biosystems).

### Haplogroup assignment and statistical analysis

MtDNA sequences were compared to the revised Cambridge Reference Sequence (rCRS) [42] using the software Geneious Pro v. 5.5.6. [43], following the phylogenetic approach [44] and the nomenclature adopted by the International Society for Forensic Genetics (ISFG) [45]. Haplogroups were assigned using Haplogrep software [46] and corrected using haplogroup assignment based on full mitochondrial genome information as performed by EMMA [47], both using Phylotree mtDNA built 16, 19 February 2014 [48]. Y chromosome haplogroups were named in accordance with Dulik et al. [49] for haplogroup Q, Trombetta et al.[50] for haplogroup E, and Karafet et al.[51] for the remaining haplogroups. The Y-STR alleles were designated according to the ISFG recommendations [52].

MtDNA sequences were submitted to EMPOP (www.empop.org) [53,54] and Y-chromosome haplotypes were submitted to YHRD (www.yhrd.org) [55] for quality control, and are available at the respective websites under accession numbers EMP00538 and YA003649 (Antioquia) and YA003818 (Cauca), respectively.

For both mtDNA and Y chromosome data, haplogroup frequencies were calculated by direct counting, diversity indices considering individuals with Native American matrilineal or patrilineal ancestry were calculated using DNAsp v. 5 [56] and Arlequin v.3.1 [57], and phylogenetic analyses were performed using Network v. 4.6.1.1 software (http://www.fluxus-engineering.com). Phylogenetic networks were sequentially constructed applying reduced median and median-joining methods. In the Y-chromosome network the DYS385 marker was not considered and the number of repeats at DYS389II was calculated after subtracting the number of repeats at DYS389I. STR weighting was applied in accordance with Qamar et al.[58] to obtain the most parsimonious network.

### Autosomal marker genotyping and analysis

All Antioquia population samples and 55 Cauca samples were additionally genotyped for the 46 AIM-InDel polymorphisms described in Pereira et al. [59], according to the single multiplex reaction described therein. These markers were selected to efficiently measure population admixture proportions from African, European, East Asian and Native American origins.

The apportionment of genetic ancestral contributions was estimated using the software STRUCTURE v2.3.3 [14,17]. To estimate the ancestral membership proportions, a supervised analysis was performed using prior information on the geographic origin of the reference samples from Africa, Europe and Native America. The STRUCTURE runs comprised three replicates of 100,000 burning steps followed by 100,000 Markov Chain Monte Carlo (MCMC) iterations. A tri-hybrid contribution from Native Americans, Europeans and Africans was assumed (i.e., K = 3). The “Use population Information to test for migrants” option was used with the Admixture model. Allele frequencies were correlated and updated using only individuals with POPFLAG = 1, (i.e., the HGDP-CEPH samples used as reference; data from Pereira et al.[59]).

## Results

MtDNA control region haplotypes observed in the studied Colombian Native American population groups, together with their classification in haplogroups, are described in detail in S1 Table. MtDNA haplogroup frequencies are indicated in Table 1 and S3 Table.

**Table 1:** mtDNA and Y chromosome major haplogroup frequencies and diversity indices in Colombian population groups.

	Department		Antioquia	Cauca		
	Group		Emberá Chamí	Cauca All	Guambiano-speaking	Chibcha-speaking
mtDNA	**Nt**		**38**	**58**	**33**	14
	**Haplogroup frequencies**	**A2**	0.474	0.190	0.090	0.429
		**B2**	0.263	0.069	0.061	0.071
		**C1**	0	0.638	0.758	0.286
		**D1**	0.263	0.086	0.091	0.143
		**L2**	0	0.017	0	0.071
	**N**		**38**	**57**	**33**	**13**
	**Diversity indices**	**K**	7	38	19	13
		**S**	22	71	45	47
		**H**	0.751 ± 0.051	0.958 ± 0.018	0.900 ± 0.043	1 ± 0.030
		**π**	0.00785 ± 0.00035	0.00931 ± 0.00069	0.00706 ± 0.00100	0.01163 ± 0.00119
Y chromosome	**Nt**		**24**	**48**	**29**	**12**
	**Haplogroup frequencies**	Q1a3*	0.167	0.188	0.310	0
		Q1a3a1a*	0.833	0.417	0.483	0.333
		**European^a^**	0	0.354	0.207	0.500
		**African^a^**	0	0.042	0	0.167
	**N**		**24**	**29**	**23**	**4**
	**Diversity indices**	**K**	13	21	16	3
		**Gene Diversity**	0.9239 ± 0.0323	0.9754 ± 0.0153	0.9644 ± 0.0224	0.8333 ± 0.2224

Diversity indices were calculated considering only Native American haplotypes. Nt stands for the total number of samples, N stands for the number of samples with a maternal or paternal Amerindian lineage that were considered for diversity indices calculation, K is the number of different haplotypes, S is the number of segregating sites, H is the haplotype diversity, π is the nucleotide diversity and MNPD is the mean number of pairwise differences.

^a^See S4 Table for details.

The male haplotypes and haplogroups detected are described in S2 Table, including the data already published in Roewer et al. [39] for the Native American haplogroups. Autosomal AIM-InDel genotypes per sample are described in S5 Table.

### MtDNA haplogroup frequencies

All individuals except one (Cauca—haplogroup L) belong to the typical Native American mtDNA haplogroups A2, B2, C1 and D1 [60].

Emberá-Chamí individuals (n = 38) belong to A2, B2 and D1 lineages (but not C), as previously reported for this ethnic group [12,21]. Within haplogroup A2 (0.474), two different sub-lineages were distinguished: A2ac at a predominant frequency (0.421), which was also described for other Colombian groups [21], and A2 (with polymorphisms 64T and 16126C) (0.053) which was also found in Emberá and Waunana groups from Panama [61]. Considering haplogroup B2 (0.263), it is noteworthy that all samples (in all linguistic sets) belong to the B2d haplogroup. Finally two different sub-lineages were found in haplogroup D (0.263): D1+16239T+16286T (0.158) that was not previously described, and D1 (0.105).

In the Cauca population (n = 58) all four typical Native American haplogroups are present, with haplogroup C1 at a highest frequency (0.638), followed by A2 (0.190), D1 (0.086) and B2 (0.069). Because this population sample is heterogeneous in what concerns the origin of its individuals (both in location and in linguistic groups), we have analysed its major speaking groups separately.

Guambiano-speaking group (n = 33) presents only Native American mtDNA haplogroups, with haplogroups A2 (0.091) and B2 (0.061) each one represented by one sub-lineage: A2ac and B2d. Within haplogroup C (0.758), the two most frequent sub-haplogroups observed were C1b (0.485) and C1d (0.273), both recognized as founder haplogroups in the Americas [60]. The majority of samples (0.394; C1b+16169T and C1b12) found within C1b also presented the 16169T polymorphism, which is also present in the Chibcha-speaking group. Within C1d three haplotypes were observed, C1d (+194T) being the most frequent (0.212). Haplogroup D1 (0.091) was represented by two haplotypes (see S3 Table for further details on the least frequent lineages).

Chibcha speaking group (n = 14) presents one African haplogroup, L2a1c1 that descends from L2a1 which is a typical African lineage in Bantu populations [62]. All four major Native American haplogroups are present in this group. A2 (0.429) is represented by 5 different haplotypes, B2d (0.071) by one, C1b (0.214) by three, C1d by one (0.071) and D1f (0.143) by two (S3 Table).

### Y chromosome haplogroup frequencies

The Y chromosome haplogroup frequencies observed in our samples are indicated in Table 1 and S4 Table.

Regarding Y chromosome haplogroups there is clearly a higher proportion of non-Native American lineages than for mtDNA, particularly in the populations from the Cauca department where various European lineages were found, in agreement with the asymmetric gender introgression pattern historically documented.

The Emberá-Chamí individuals from Antioquia present only Native American haplogroups, with the M3 derived linage Q1a3a1a* at a higher frequency (0.833) than the M3 ancestral Q1a3* (0.167).

In the Cauca populations, Native American (0.604), European (0.354), and sub-Saharan African (0.042) haplogroups were found.

The Guambiano-speaking group shows both the Q1a3a1a*-M3 (0.483) and the Q1a3*-M346 (0.310) lineages at similar proportions, and the remaining 0.207 of the Y chromosomes belong to the European haplogroups R1b1-P25 and J2-M172.

In the Chibcha-speaking group the Native American lineage Q1a3*-M346 is absent, and only four individuals present the M3 derived lineage Q1a3a1a* (0.333), while the non-Native American linages are present at a higher frequency (0.667) and represented by haplogroups R1b1-P25, G-M201, J2-M172, E1b1b1a1-M78 and E1b1b1c-M123 (0.500) that are typical in Europeans, and by two samples from the sub-Saharan African E1b1a1*-M2 haplogroup (0.167). Despite its small sample size, Sub-Saharan haplogroups were detected only in the Chibcha group, similarly to the results obtained for mtDNA.

### Diversity Indices

Maternal or paternal diversities were calculated only within lineages of Native American ancestry (Table 1).

MtDNA diversity indices showed generally higher values in the Cauca samples than in the Emberá-Chamí. While the Emberá-Chamí population presents only 7 different haplotypes in 38 samples (H = 0.751±0.051), the 57 Cauca individuals presented 38 different haplotypes (H = 0.958 ±0.018). Both major speaking-groups from Cauca present higher diversity values than the Emberá-Chamí population: the Guambiano-speaking group presents 19 different haplotypes in 33 individuals (H = 0.900±0.043), and the Chibcha-speaking group is highly diverse with 13 different haplotypes in 13 individuals (H = 1±0.030).

Because the Chibcha sample presented few male individuals with Native American Y lineages (N = 4), the diversity indices for this population should be regarded with caution. For the other populations, however, the Y chromosome diversity indices follow the same trend than that described for mtDNA. The Emberá-Chamí population presents lower gene diversity (0.924±0.032) than the Guambiano (0.964±0.022) or the Cauca population considered as a whole (0.975±0.015), and the same is observed for the number of different haplotypes: 13 in 24 individuals for the Emberá-Chamí and 16 in 23 individuals for the Guambiano-speaking group. Despite this observation, the Emberá-Chamí population presents a slightly higher mean number of pairwise differences (MNPD) (9.214±4.390) than the Guambiano-speaking group (8.075±3.891) or the total Cauca population (8.325±3.971).

### Phylogenetic analysis

**Fig 2:**
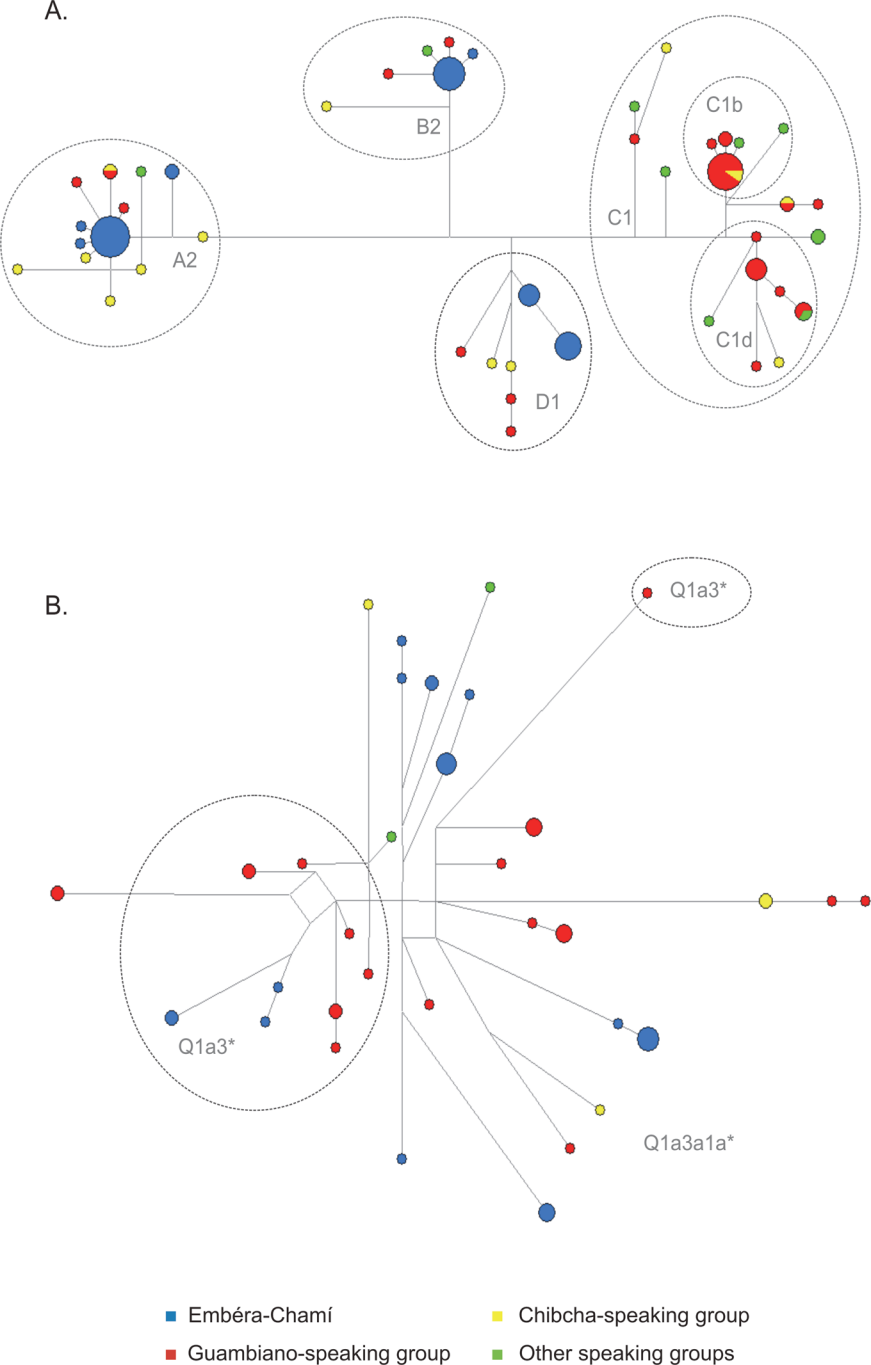
Phylogenetic network of the Native American mtDNA (a) and Y chromosome (b) haplotypes detected in this study. Circle size is proportional to the number of haplotypes and branch size is proportional to the number of polymorphisms that distinguish each pair of haplotypes. Dashed lines delimit different haplogroups.

The network analysis shows clearly the presence of the four typical mtDNA Native American lineages in the Colombian populations (Fig. 2A), with the Antioquia Emberá-Chamí population presenting only 5 mtDNA founder haplotypes while the Cauca populations present a higher number of lineages. In the Y chromosome network (Fig. 2B) the same differences between populations can be visualised, although the lineages are more diverse than in the mtDNA.

It is also noticeable that, for both mtDNA and Y chromosome, no haplotypes are shared between Antioquia and Cauca groups, although in each group there are various haplotype clusters distant from each other. This observation, together with the values of MNPD described above, is probably the result of bottleneck events acting on genetically differentiated and somewhat isolated Native American groups, as well as of the small sizes of the samples analysed.

### Autosomal AIM-InDel results and comparison with lineage markers

The genotyping results obtained for the 46 AIM-InDels are listed in S5 Table. These data were used to calculate the Native American (NAM), European (EUR) and African (AFR) contributions using STRUCTURE software [14,17], as detailed in M&M. Admixture proportions obtained for mtDNA, Y chromosome and AIM-InDels are compared in Table 2. The average value between lineage markers ancestries and the ancestry value obtained for the autosomal markers were compared to check for multiple events of sex-biased gene flow, as reported previously for some Colombian admixed populations [25,28]. This trend is clear in admixed populations but not in NAM populations like Cauca. Although the Cauca sample presents almost 100% of NAM mtDNA haplotypes, when averaging the proportions obtained for mtDNA and Y chromosome, the values obtained are very similar to those observed for the autosomal AIM-InDels. In the Antioquia sample, while both lineage markers presented 100% NAM ancestry, the autosomal AIM-InDels present ~3% EUR ancestry and ~2% AFR ancestry. These values may be considered residual non-NAM ancestry that could only be detected with the analysis of the autosomal InDels due to the higher number of unlinked markers studied in twice the number of chromosomes analysed in the non-recombining portions of the genome.

**Table 2:** Native American (NAM), European (EUR) and African (AFR) admixture proportions in the Colombian samples from the departments of Cauca and Antioquia.

	Cauca				Antioquia			
	n	NAM	EUR	AFR	n	NAM	EUR	AFR
mtDNA	58	98.30%	0.00%	1.70%	38	100.00%	0.00%	0.00%
Y chromosome	48	60.42%	35.42%	4.17%	24	100.00%	0.00%	0.00%
Average between lineage markers		79.36%	17.71%	2.94%		100.00%	0.00%	0.00%
AIM-InDels	110	77.72%	17.48%	4.81%	76	94.96%	3.32%	1.71%

n stands for the number of chromosomes analysed

## Discussion

The study of mtDNA, Y chromosome and autosomal AIM-InDel data in these groups contributes to a better knowledge of the genetic composition of Colombian populations.

We show several important differences between the two samples, including different genetic composition and different degrees of admixture. Indeed, while the proportion of NAM matrilineal ancestry is well preserved in all samples, the proportion of paternal influx differed widely among samples, as reflected in the AIM-InDel data. The Emberá-Chamí sample from Antioquia presented exclusively Native American haplogroups both in mtDNA and Y chromosome, and only a residual proportion of non-Native ancestry in the autosomal AIM-InDels. The Cauca sample revealed a significant non-NAM male input (0.396), particularly within the Chibcha-speaking group, with its mtDNA haplogroups being almost exclusively NAM (only one AFR haplotype was detected), which reflects the traditional sex biased admixture between NAM and EUR men [12,13,25]. This sample harbours a proportion of EUR and AFR autosomal ancestry that is equivalent to the average between the values obtained for the lineage markers, in contrast to what was recently observed for Colombian Mestizo groups from the same departments [28], suggesting no important sex biased admixture events occurred recently. The Emberá-Chamí sample demonstrates a strikingly different profile due to the complete absence of non-NAM lineages in both lineage markers analysed, an observation that is rare in extant NAM populations, particularly in what concerns the paternal lineages. Consistently, it also presents lower diversity values (particularly a small number of different haplotypes) than the Cauca populations, which may be justified by a relatively recent bottleneck event caused by a decrease in the effective population size during the Spanish colonization; despite this, it still retains representatives of 3 of the different major NAM mtDNA lineages and of 2 different NAM Y chromosome haplogroups.

It is also noteworthy that the groups with a higher degree of Y chromosome non-NAM input show also a higher NAM mtDNA diversity. Although we can not exclude that larger Native populations may have attracted more non-Native admixture, we also suggest that populations exposed to a higher European admixture were less subjected to a decrease in effective population sizes and thus their Native non-recombining lineages were less prone to be erased by genetic drift. This protective effect of the admixture events on the genetic diversity of uniparental lineages is clear in the populations from Cauca, a less isolated and more cosmopolitan region than the Emberá-Chamí population from Antioquia.

The study of the Native American genetic diversity has been complicated by the fact that, although the more isolated populations retain the Native American genetic identity, they paid the cost of losing most of its diversity by the decrease in effective population sizes after the European colonization. However this study demonstrates that, for lineage markers, it is possible to recover more of the Native American genetic diversity from the study of a comprehensive sampling of Native American ancestry individuals in admixed populations than from the analysis of isolated communities.

## Supporting Information

S1 Table mtDNA haplotypes. (XLSX)

S2 Table Y chromosome data. (XLSX)

S3 Table Frequencies of mtDNA sublineages. (XLSX)

S4 Table Y chromosome haplogroup frequencies. (XLSX)

S5 Table Autosomal AIM-InDels data. (XLSX)

S6 Table Population density of the sampled Cauca municipalities. (XLSX)

S1 Fig Phylogenetic tree of Y-haplogroups analyzed in the present study. The haplogroups are named in accordance with Karafet et al.[51].upgraded by Trombetta et al.[50] and Dulik et al.[49] for haplogroups E and Q, respectively.
(DOCX)
