# Supplementary material for: Admixture and Genetic Diversity Distribution Patterns of Non-Recombining Lineages of Native American Ancestry in Colombian Populations
Source: PLoS One. 2015 Mar 16;10(3):e0120155. doi: 10.1371/journal.pone.0120155 (PMC4361580; doi:10.1371/journal.pone.0120155)
Supplement: S1 Fig — The haplogroups are named in accordance with Karafet et al.[51].upgraded by Trombetta et al.[50] and Dulik et al.[49] for haplogroups E and Q, respectively. (DOCX) [file pone.0120155.s007.docx]

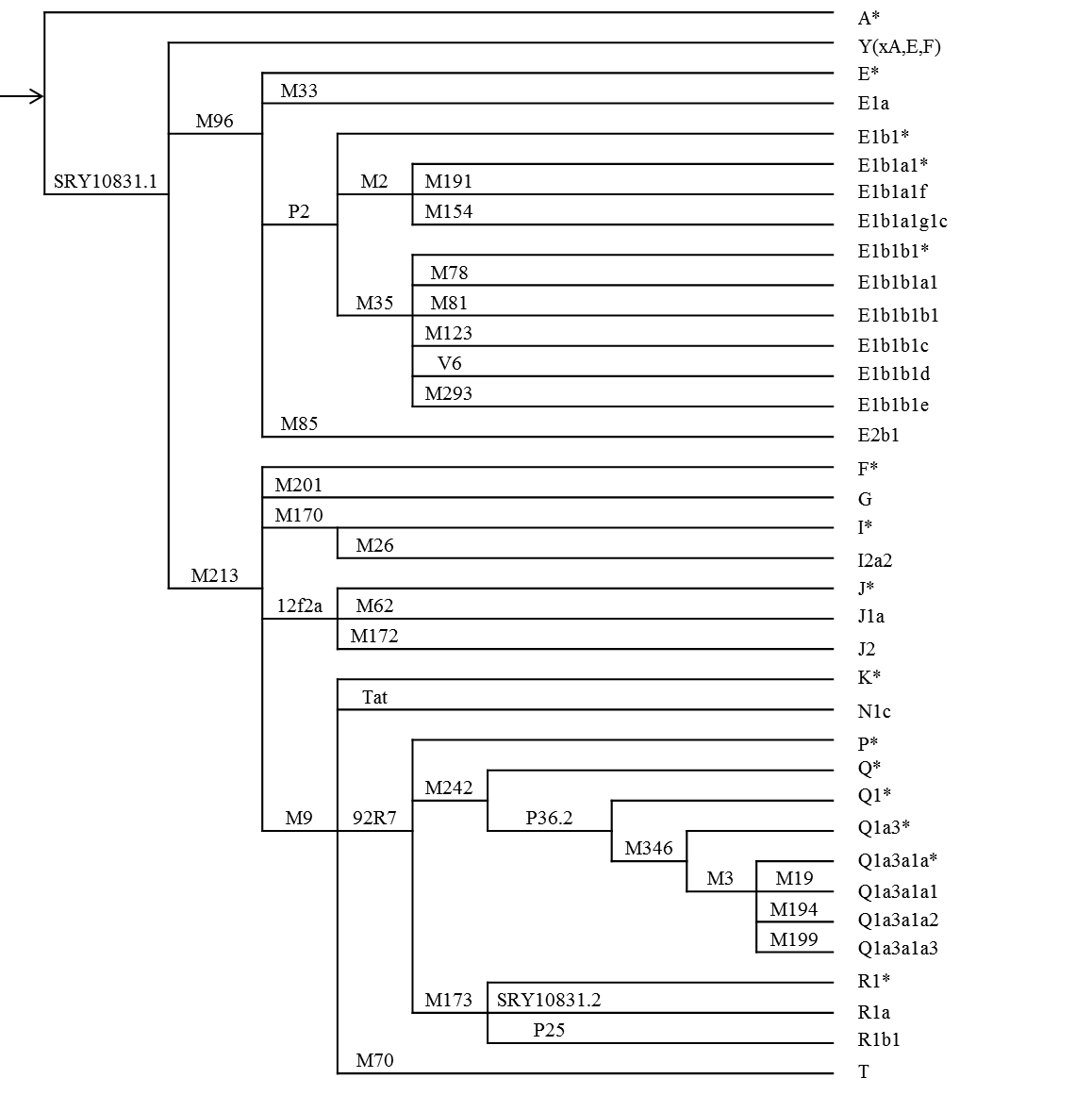


Fig.S1 Phylogenetic tree of Y-haplogroups analyzed in the present study. The haplogroups are named in accordance with Karafet et al. [[1](#_ENREF_1)] upgraded by Trombetta et al. [[2](#_ENREF_2)] and Dulik et al. [[3](#_ENREF_3)] for haplogroups E and Q, respectively.

1. Karafet TM, Mendez FL, Meilerman MB, Underhill PA, Zegura SL,Hammer MF (2008) New binary polymorphisms reshape and increase resolution of the human Y chromosomal haplogroup tree. Genome Res 18 (5):830-838. doi:gr.7172008 [pii]

10.1101/gr.7172008

2. Trombetta B, Cruciani F, Sellitto D,Scozzari R (2011) A new topology of the human Y chromosome haplogroup E1b1 (E-P2) revealed through the use of newly characterized binary polymorphisms. PLoS One 6 (1):e16073. doi:10.1371/journal.pone.0016073

3. Dulik MC, Owings AC, Gaieski JB, Vilar MG, Andre A, Lennie C, Mackenzie MA, Kritsch I, Snowshoe S, Wright R, Martin J, Gibson N, Andrews TD, Schurr TG,Consortium TG (2012) Y-chromosome analysis reveals genetic divergence and new founding native lineages in Athapaskan- and Eskimoan-speaking populations. Proceedings of the National Academy of Sciences 109 (22):8471-8476. doi:10.1073/pnas.1118760109
